# Seropositive polyarthritis and diffuse lymphadenopathy associated with *PRKCD* mutation

**DOI:** 10.1093/rheumatology/kead639

**Published:** 2023-12-08

**Authors:** Riccardo Papa, Francesca Schena, Anna Corcione, Paola Bocca, Enrico Drago, Clara Malattia, Alice Grossi, Isabella Ceccherini, Marco Gattorno

**Affiliations:** UOC Reumatologia e Malattie Autoinfiammatorie, IRCCS Istituto Giannina Gaslini, Genoa, Italy; UOC Reumatologia e Malattie Autoinfiammatorie, IRCCS Istituto Giannina Gaslini, Genoa, Italy; UOC Reumatologia e Malattie Autoinfiammatorie, IRCCS Istituto Giannina Gaslini, Genoa, Italy; UOC Reumatologia e Malattie Autoinfiammatorie, IRCCS Istituto Giannina Gaslini, Genoa, Italy; UOC Reumatologia e Malattie Autoinfiammatorie, IRCCS Istituto Giannina Gaslini, Genoa, Italy; UOC Reumatologia e Malattie Autoinfiammatorie, IRCCS Istituto Giannina Gaslini, Genoa, Italy; Laboratory of Genetics and Genomics of Rare Diseases, IRCCS Istituto Giannina Gaslini, Genoa, Italy; Laboratory of Genetics and Genomics of Rare Diseases, IRCCS Istituto Giannina Gaslini, Genoa, Italy; UOC Reumatologia e Malattie Autoinfiammatorie, IRCCS Istituto Giannina Gaslini, Genoa, Italy

Rheumatology key message

- Heterozygous *PRKCD* p.Gly248Ser mutation may favor early-onset autoimmunity and lymphoproliferation.

Dear Editor, Juvenile idiopathic arthritis (JIA) is a diagnosis of exclusion that encompasses all forms of chronic arthritis of unknown origin with onset before the age of 16 years [1]. Increasing evidence supports a genetic background [2]. Protein kinase C (PKC) δ is a serine threonine kinase involved in cell proliferation, differentiation and apoptosis whose deficiency is associated with early-onset autoimmunity and autosomal recessive autoimmune lymphoproliferative syndrome, type III (OMIM #615559) [3, 4]. Patients with homozygous mutations of the *PRKCD* gene, coding for PKCδ, show laboratory lymphocytes defects that are even reported in heterozygous asymptomatic parents [4]. Attenuated or late-onset phenotypes in heterozygotes have been supposed [4].

We describe a patient carrying a heterozygous *PRKCD* mutation associated with lymphocytes defects developing a rheumatoid factor-positive polyarticular JIA during pubertal age. Informed consent was provided for the publication of this article.

The 18-year-old girl was born from unrelated healthy parents. Since 6 years of age, she has displayed self-resolving episodes of erythema nodosum and early puberty, requiring triptorelin for 4 years. At 12 years of age, she presented with low-grade fever and polyarthritis involving knee, ankle, metatarsophalangeal and interphalangeal joints, associated with high acute phase reactants, positive rheumatoid factor and mild anaemia at laboratory tests. A panel of autoantibodies was negative except for anti-tropomyosin antibodies and lupus anticoagulant. Whole-body MR short time inversion recovery images and TC scan showed diffuse lymphadenopathy (Fig. 1A–C, respectively). The bone marrow aspirate was normal. Oral steroids obtained only a temporary benefit and intra-articular steroidal injections, associated with subcutaneous etanercept and methotrexate, were administered, achieving complete remission. After two years, methotrexate was gradually tapered within 6 months. The following year, during etanercept tapering, she displayed severe reactivation of polyarticular inflammation, associated with brachial biceps tendonitis and calf fasciitis (Fig. 1D and E). Laboratory tests showed rising inflammatory markers (Fig. 1F) and positive rheumatoid factor. Etanercept and methotrexate were re-started at full dosage and a second administration of intra-articular steroidal injections was required for clinical and laboratory remission.

**Figure 1.**
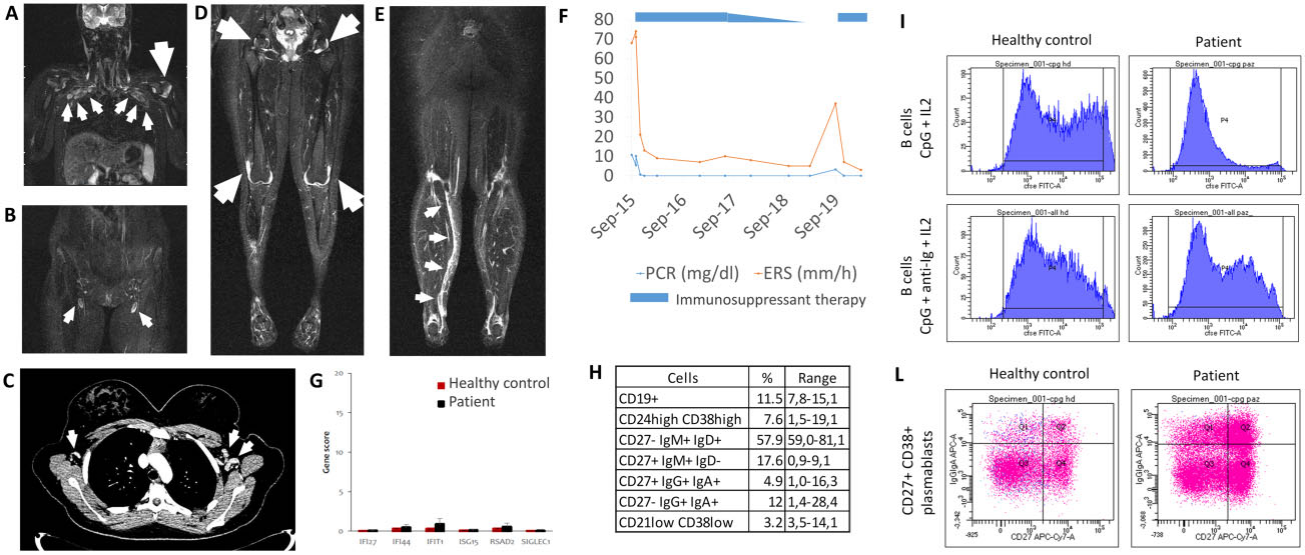
Imaging and laboratory features of the patient. Short time inversion recovery MR images (**A**, **B**) and TC scan (**C**) showing diffuse lymphadenopathy and joint effusion (white arrow) at the disease onset. Polyarticular effusions (**D**, white arrows) and calf fasciitis (**E**, white arrows) at the short time inversion recovery MR and increased laboratory tests (**F**) during treatment tapering. Negative type I interferon signature (**G**) and increased IgM memory B cells (**H**) at the disease flare. Increased B-cell proliferation after CpG and interleukin (IL)-2 stimulation (**I**) and percentage of plasmablasts (**J**) compared with a healthy control

Due to the atypical onset of diffuse lymphadenopathy in a child with polyarticular JIA, a next generation sequencing panel of genes associated with autoimmune lymphoproliferative syndrome was performed, revealing the heterozygous c.742G>A, p.Gly248Ser missense mutation of *PRKCD*, later confirmed by Sanger sequencing. Analysis of the parents showed *de novo* inheritance. The mutation is absent from the internal database and present in five heterozygotes in the Genome Aggregation Database (available at http://gnomad.broadinstitute.org) and one homozygote has been reported [5]. All common prediction software for pathogenicity and conservation suggest damaging and highly conservative scores, with a Genomic Evolutionary Rate Profiling rejected substitution score of 5.16 (range from –12.3 to +6.17). Based on this evidence, the variant is presently classified as of unknown significance/likely pathogenic (see https://varsome.com/).

To investigate a possible role of the *PRKCD* mutation in the patient phenotype, we performed a detailed immune workup, revealing high levels of serum immunoglobulin (Ig) isotype M with normal concentration of IgG and IgA and normal total lymphocytes count. The type I interferon signature was negative (Fig. 1G). B-cell subpopulation analysis revealed an increased concentration of IgM memory B cells (Fig. 1H) and B-cell proliferation in response to Toll-like receptor 9 agonist was markedly increased (Fig. 1I) as well as the percentage of plasmablasts (Fig. 1L). T-cell subpopulations were normal, including the double negative T cells. A defective Fas-mediated T-cell apoptosis test was detected (data not shown). These immunological features clearly recall the lymphocytes defect described in symptomatic carriers of the homozygous *PRKCD* mutation.

The present case suggests a possible role of the heterozygous PRKCD p.Gly248Ser mutation in a child with an unusual phenotype consisting of seropositive polyarticular JIA, diffuse lymphoproliferation and lymphocytes defect. The substitution of glycine within the conserved one regulatory domain of the protein may alter the diacylglycerol/phorbitol ester-induced activation of PKCδ, causing increased transcription of a dysfunctional protein, as recently demonstrated [5].Thus, contrary to other *PRKCD* mutations usually associated with PKCδ deficiency, the p.Gly248Ser mutation may affect the PKCδ pathway even at the heterozygous state, causing a milder deficiency of B-cell differentiation and T-cell apoptosis, with consequent increased proliferation of autoreactive B-cell clones secreting autoantibodies. Further evidence will be required to confirm this clinical observation. We suggest including *PRKCD* in genetic tests in patients with JIA associated with diffuse lymphadenopathy.

## Data Availability

The data underlying this article will be shared on reasonable request to the corresponding author.
